# Synthesis of Cu_2_O/CuO Nanocrystals and Their Application to H_2_S Sensing

**DOI:** 10.3390/s19010211

**Published:** 2019-01-08

**Authors:** Kazuki Mikami, Yuta Kido, Yuji Akaishi, Armando Quitain, Tetsuya Kida

**Affiliations:** 1 Department of Applied Chemistry and Biochemistry, Graduate School of Science and Technology, Kumamoto University, Kumamoto 860-8555, Japan; kazuki.mikami@furukawaelectric.com (K.M.); kido.yuta.63@gmail.com (Y.K.); 183d8801@st.kumamoto-u.ac.jp (Y.A.); 2 College of Cross-Cultural and Multidisciplinary Studies, Kumamoto University, Kumamoto 860-8555, Japan; quitain@kumamoto-u.ac.jp; 3 Faculty of Advanced Science and Technology, Kumamoto University, Kumamoto 860-8555, Japan

**Keywords:** gas sensor, nanocrystal, Cu_2_O, CuO, H_2_S

## Abstract

Semiconducting metal oxide nanocrystals are an important class of materials that have versatile applications because of their useful properties and high stability. Here, we developed a simple route to synthesize nanocrystals (NCs) of copper oxides such as Cu_2_O and CuO using a hot-soap method, and applied them to H_2_S sensing. Cu_2_O NCs were synthesized by simply heating a copper precursor in oleylamine in the presence of diol at 160 °C under an Ar flow. X-ray diffractometry (XRD), dynamic light scattering (DLS), and transmission electron microscopy (TEM) results indicated the formation of monodispersed Cu_2_O NCs having approximately 5 nm in crystallite size and 12 nm in colloidal size. The conversion of the Cu_2_O NCs to CuO NCs was undertaken by straightforward air oxidation at room temperature, as confirmed by XRD and UV-vis analyses. A thin film Cu_2_O NC sensor fabricated by spin coating showed responses to H_2_S in dilute concentrations (1–8 ppm) at 50–150 °C, but the stability was poor because of the formation of metallic Cu_2_S in a H_2_S atmosphere. We found that Pd loading improved the stability of the sensor response. The Pd-loaded Cu_2_O NC sensor exhibited reproducible responses to H_2_S at 200 °C. Based on the gas sensing mechanism, it is suggested that Pd loading facilitates the reaction of adsorbed oxygen with H_2_S and suppresses the irreversible formation of Cu_2_S.

## 1. Introduction

Copper oxides (Cu_2_O or CuO) are among the important oxide materials because of their versatile functionalities. Their low cost and toxicity are advantageous for commercial uses. Major applications of copper oxides in chemistry include catalysis [1], solar cells [2,3], batteries [4,5,6], and, gas sensors [7,8,9]. For such applications, copper oxide nanostructures such as nanoparticles, nanocrystals, nanorods, nanocubes, nanosheets, etc. have been extensively used to enhance performance [10].

The control of crystal size is very important for resistive-type gas sensors using oxide materials. It has been reported that the reduction of the crystal size of oxides into a nanosize regime drastically improves the gas sensing properties [11,12]. This effect is explained in terms of the effective formation of electron-depleted regions in nanosized crystals by oxygen adsorption, which induces a significant change in electrical conductivity upon gas reaction [13,14]. Thus, the use of oxide nanoparticles and nanocrystals is one of the most efficient ways to develop high-performance gas sensors [15,16]. There have been many reports on the gas sensing properties of CuO nanoparticles deposited on other semiconductors such as SnO_2_ [17], ZnO [18], graphene oxide [19]. Thin film CuO gas sensors have recently been well-reviewed [20]. However, few studies have investigated the gas sensing properties of CuO and Cu_2_O nanoparticles/nanocrystals [21,22,23].

So far, several routes have been developed to synthesize nanoparticles and nanocrystals of copper oxides, including precipitation [24,25], sonochemical [26], microwave irradiation [27], thermal decomposition [28], solid-state reaction [29], and other methods [30,31,32]. In this study, to produce high quality copper oxide nanocrystals, we focused on a hot-soap method in which metal precursors are dissolved in a high-boiling-point organic solvent with surface coordinating ligands to produce metal-ligand complexes, which are then decomposed at elevated temperature [33,34]. This methodology was first developed to synthesize quantum dots (semiconductor nanocrystals) with high photoluminescent quantum yields [35,36,37]. Using the hot-soap method, it is easy to synthesize highly crystalline spherical nanocrystals with a narrow size distribution.

Here, we attempted to produce monodispersed Cu_2_O and CuO nanocrystals by a hot-soap method (heating-up method) and examined their gas sensing properties. The synthesis of Cu_2_O nanocrystals by hot-soap methods has already been reported [23,38]. However, we found that phase-pure Cu_2_O nanocrystals of approximately 12 nm were readily synthesized by simply heating up a mixture containing copper precursors and diol in oleylamine. The conversion into CuO nanocrystals was also achieved by oxidation with atmospheric air at room temperature. The H_2_S sensing capability of the copper oxide (Cu_2_O and CuO) nanocrystals was evaluated by fabricating thin film gas sensor devices. The effects of operating temperature and Pd loading on the sensor performance were examined to clarify the sensing mechanism and to improve the sensing properties.

## 2. Materials and Methods

### 2.1. Cu_2_O and CuO Nanocrystal Synthesis

Cu_2_O nanocrystals (NCs) were synthesized by a heating-up method using oleylamine as a high-boiling-point solvent, which also works as a coordinating ligand that suppresses the crystal growth. Typically, Cu (II) acetylacetonate (Cu(acac)_2_) (1 mmol) and 1,8-octanediol (1 mmol) were added to oleylamine (15 mL) in a three-necked flask. The flask was connected to a Schlenk line, and the reaction system was heated at 80 °C for 30 min under vacuum to remove water and oxygen from the system. Then, the temperature was raised to 160 °C under an Ar flow with a heating rate of 2 °C/min and kept at 160 °C for 60 min. After the reaction, the obtained NCs were washed with a mixture of hexane and isopropanol several times by centrifugation. The NCs were dispersed in toluene to produce a coating ink (0.2 g/L). CuO NCs were synthesized by air oxidation. The Cu_2_O NCs were stored in toluene under atmospheric air for a week, converting them to CuO NCs.

### 2.2. Pd Nanocrystal Synthesis

Pd NCs were also synthesized by a heating-up method according to the method reported by Kim et al. [39]. Pd acetate (0.43 mmol) was dissolved in trioctylphosphine (1 mL) in a three-necked flask. The mixture was stirred at 80 °C for 30 min under an Ar flow for 15 min, followed by addition of oleylamine (10 mL). Then, the temperature was raised to 200 °C under an Ar flow with a heating rate of 2 °C/min and kept at 160 °C for 60 min. After the reaction, the obtained NCs were washed with a mixture of hexane and isopropanol several times by centrifugation at 15,000 rpm. The NCs were dispersed in toluene to produce a colloidal suspension (0.1 g/L).

### 2.3. Material Characterization

The synthesized NCs were analyzed by X-ray diffraction (XRD) using Cu Kα radiation (MiniFlex600; Rigaku, Tokyo, Japan) and transmission electron microscopy (TEM) (JEM-2000X; JEOL, Tokyo, Japan). The size of colloid particles was determined with a dynamic light scattering (DLS) spectrophotometer (Zetasizer Nano ZS; Malvern Instruments, Malvern, Worcestershire, UK). The presence and absence of capping agents on NCs were determined by Fourier transform-infrared (FT-IR) spectroscopy (FTIR4100; JASCO, Tokyo, Japan). Light absorbance spectra were acquired with a UV-vis spectrometer (V-650; JASCO).

### 2.4. Sensor Fabrication and Sensing Measurements

**Figure 1:**
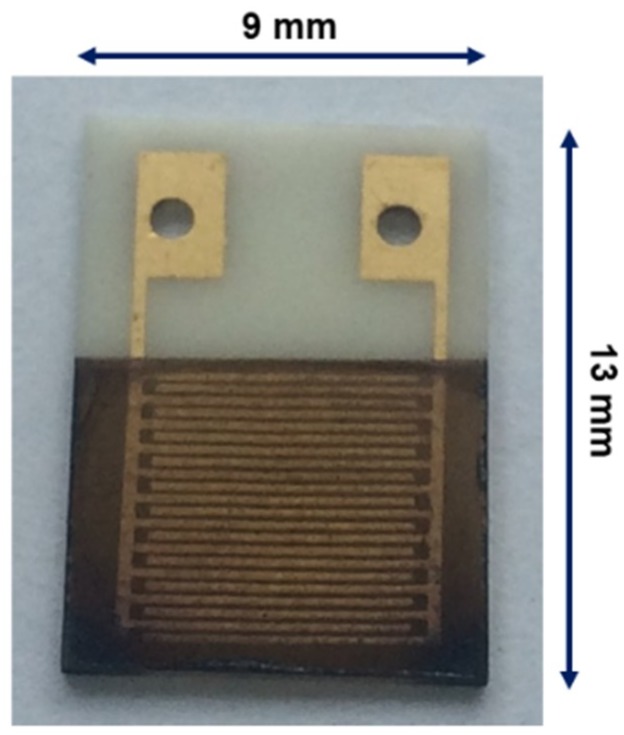
A photo of the sensor device using Cu_2_O NCs deposited on an alumina substrate with Au microelectrodes.

A pair of comb-type Au microelectrodes (line width: 180 μm; distance between lines: 90 μm; sensing layer area: 70 mm^2^) was formed on an alumina substrate (9 × 13 × 0.38 mm) by a screen-printing method. A commercial Au paste (AU-176010; Nilaco, Tokyo, Japan) was coated through a screen mesh on an alumina substrate that was cleaned by H_2_O_2_ and NH_3_ solutions heated at 80 °C. The coated Au paste pattern was heated at 850 °C for 3 h. The suspension (0.1 g/L) was coated on the alumina substrate equipped with the Au microelectrodes by spin coating at 1500 rpm to fabricate gas sensing films of approximately 150 nm thickness. To fabricate Pd-loaded copper oxide NC films, the copper oxide NC suspension was mixed with a designated amount of the Pd NC suspension, and then the mixture was deposited on the alumina substrate by spin coating. The Pd loading amount was controlled to 1, 5, and 10 mol%, relative to the copper oxide amount. A photo of the device is shown in Figure 1.

The sensing properties of the fabricated devices were examined using a conventional gas flow apparatus. The flow rates of sample gases were set to 100 cm^3^/min with mass flow controllers (SEC-series; HORIBA STEC, Kyoto, Japan). Sample gases of H_2_S in air (21% O_2_ in N_2_) were prepared by diluting parent H_2_S in nitrogen with synthetic nitrogen and oxygen. The parent synthetic gas mixture was purchased from Taiyo Nippon Sanso, Tokyo, Japan. A sensor device was connected with a standard resistor in series, and the voltage across the standard resistor was measured under an applied voltage of DC 4 V to measure the electrical resistance of the devices. The sensor was externally heated with an electric furnace at 50–250 °C. The electrical signal of the sensor was acquired with a multimeter (2701; KEITHLEY, Beaverton, OR, USA). The sensor response (S) was defined as the ratio of electrical resistance in air containing H_2_S (R_g_) to that in air (R_a_) (S = R_g_/R_a_).

## 3. Results and Discussion

### 3.1. Characterization of Copper Oxide NCs

**Figure 2:**
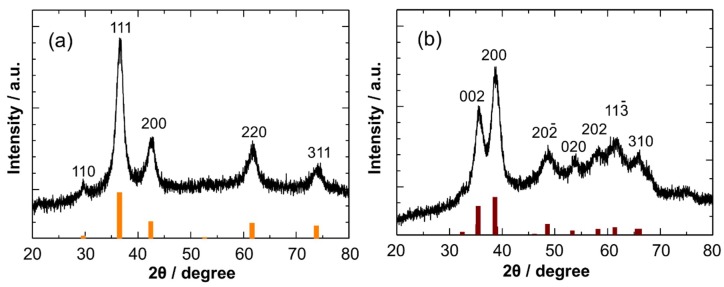
XRD patterns of (**a**) Cu_2_O (JCPDS file no. 05-0667) and (**b**) CuO NCs (JCPDS file no. 45-0937). CuO NCs were obtained by air oxidation of Cu_2_O NCs at room temperature.

Figure 2a shows an XRD pattern of Cu_2_O NCs. The obtained pattern matched well with that for Cu_2_O without detectable impurities. The broad XRD peaks are a clear indication of the formation of NCs. The crystallite size was determined to be 4.9 nm from the Scherrer’s equation. The air oxidation of the Cu_2_O NCs led to the formation of CuO, as confirmed in Figure 2b: the XRD pattern completely changed into that of CuO. The broad XRD peaks are maintained after the phase transformation. The crystallite size was estimated to be 4.4 nm, indicating that the crystallite size did not change significantly even after air oxidation.

**Figure 3:**
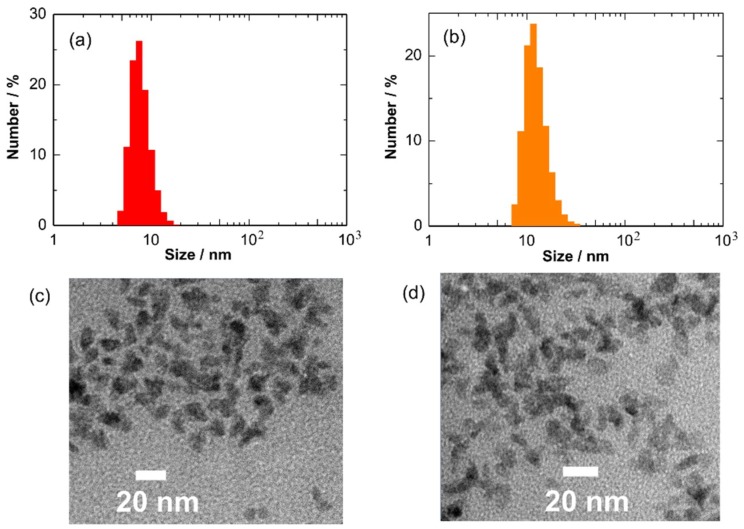
Particle size distributions dispersed in toluene and TEM images of (**a**,**c**) Cu_2_O and (**b**,**d**) CuO NCs.

Figure 3a,b shows particle size distributions of Cu_2_O and CuO NCs in toluene. The colloidal size of Cu_2_O NCs was determined to be 12.7 nm in average. The Cu_2_O NCs showed a good monodispersity in toluene. However, the colloidal size was larger than the crystallite size, suggesting the aggregation of NCs in toluene. The air oxidation of Cu_2_O NCs increased the colloidal size to 23.9 nm. One possible reason is the partial removal of hydrophobic oleylamine ligands from the NC surface after washing, leading to a decrease in solubility in toluene. Figure 3c,d shows the TEM images of the Cu_2_O and CuO NCs stored in toluene. The TEM image indicates the formation of rather irregular-shaped NCs. The particle sizes of Cu_2_O and CuO NCs were estimated to be within the range of 7 to 14 nm and 10 to 33 nm, respectively, confirming the aggregation of NCs after storage for a week. The TEM results are in good accordance with the DLS results.

**Figure 4:**
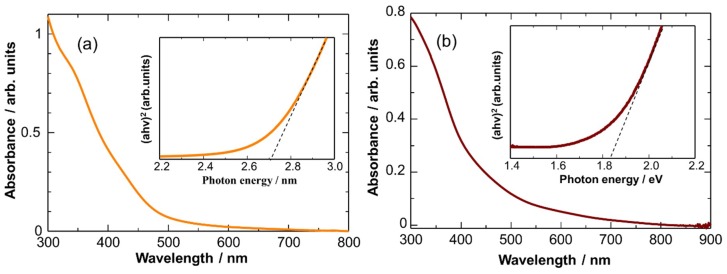
UV-vis absorption spectra of (**a**) Cu_2_O NCs and (**b**) CuO NCs in toluene. Insets show the corresponding Tauc plots.

To collect evidence of the formation of CuO NCs after air oxidation, their UV-vis absorption properties were characterized. Figure 4 shows UV-visible absorbance spectra of the Cu_2_O and CuO NCs in toluene with the corresponding Tauc plots ((αhν)^n^ vs. hν, n = 2). Obviously, the light absorption edge of the CuO NCs was red shifted as compared with that of the Cu_2_O NCs. The band gaps of the Cu_2_O and CuO NCs were calculated to be ca. 2.7 and 1.8 eV, respectively, from the Tauc plots. These values are larger than the reported values of bulk Cu_2_O (2.4 eV) and CuO (1.4 eV) [40], probably due to the quantum confinement effect. The linear fitting of (αhν)^2^ with the photon energy at n = 2 is in good agreement with the fact that Cu_2_O and CuO are direct band gap semiconductors. All of the above characterizations indicate the formation of monodispersed Cu_2_O and CuO NCs by the simple heating-up method and subsequent air oxidation, respectively.

**Figure 5:**
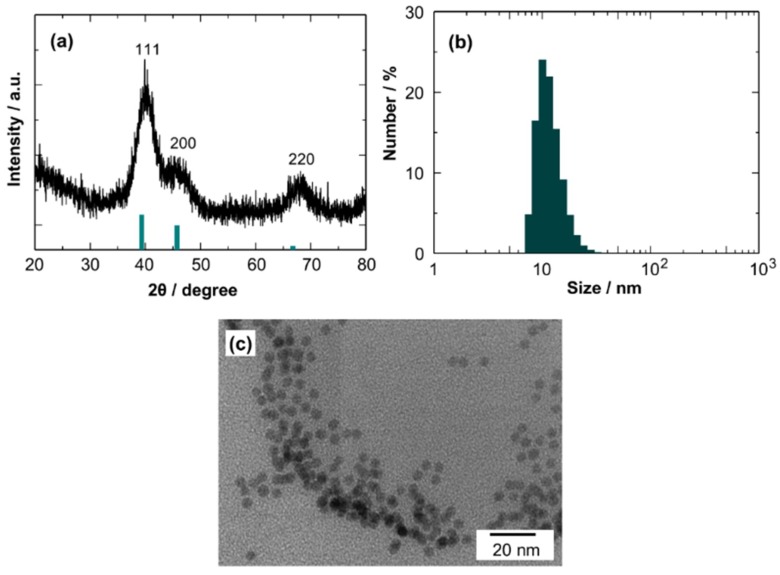
(**a**) XRD pattern, (**b**) particle size distribution, and (**c**) TEM image of Pd NCs.

Figure 5 shows the characterization results of Pd NCs. The XRD pattern matches well with that for Pd (JCPDS file no. 46-1043). The determined crystallite size is 3.8 nm. The average colloidal size of the Pd NCs in toluene was estimated to be 11 nm by DLS measurements, suggesting a slight aggregation of NCs in toluene. The TEM images revealed the formation of monodispersed spherical Pd NCs of 3.7 to 4.2 nm in diameter, which is in good agreement with the crystallite size. The results clearly show that monodispersed Pd NCs were also obtained by the simple heating-up method.

### 3.2. Thermal Treatment of Copper Oxide NCs

The use of capping agents in a heating-up method is of critical importance in the synthesis of NCs with narrow size distribution. Capping agents prevent crystal nuclei from growing abruptly and irregularly, producing monodispersed nanocrystals. The hydrophobic surface-adsorbed capping agents also make NCs well-dispersible in nonpolar solvents. However, capping agents on the NC surface behave as an insulating layer and block charge transport. Thus, the removal of capping agents is necessary for device applications of NCs that are synthesized using the present method. Several routes have been developed for stripping of capping agents from the NC surface [41,42,43,44]. Here, we attempted to remove surface-adsorbed oleylamine by a simple heat treatment at a relatively lower temperature. We also studied the phase transition of the Cu_2_O NCs by heat treatment.

**Figure 6:**
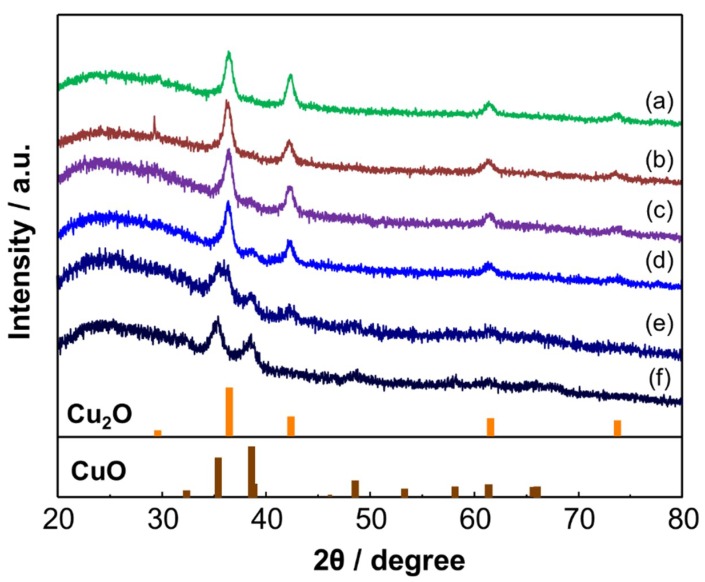
XRD patterns of Cu_2_O NCs heated at (**a**) 50, (**b**) 100, (**c**) 150, (**d**) 200, (**e**) 250, and (**f**) 300 °C for 30 min in air. Cu_2_O: JCPDS file no. 05-0667, CuO: JCPDS file no. 45-0937.

The phase stability of the Cu_2_O NCs was examined by XRD. For the analyses, the Cu_2_O NCs were deposited on a glass substrate to form a NC film. Figure 6 shows XRD patterns of the Cu_2_O NCs after heat treatment in air at different temperatures for 30 min. The Cu_2_O phase was clearly observed at 50–200 °C. However, peaks ascribable to CuO appeared at 200 °C. The phase conversion of Cu_2_O to CuO was almost complete at 300 °C. It was also confirmed that the crystallite size of the Cu_2_O NCs did not significantly change from the original size by the thermal treatment, i.e., within the range of 4–10 nm, showing their good thermal stability against crystal growth. To examine the thermal stability of the Cu_2_O phase in more detail, the time dependent XRD patterns were measured at 150 and 200 °C, as shown in Figure S1. It was confirmed that the Cu_2_O phase was stable at 150 °C; the peaks ascribable to CuO were not seen in the pattern even after heating at 150 °C for 12 h. In contrast, the CuO phase began to appear after heating at 200 °C for 2 h.

**Figure 7:**
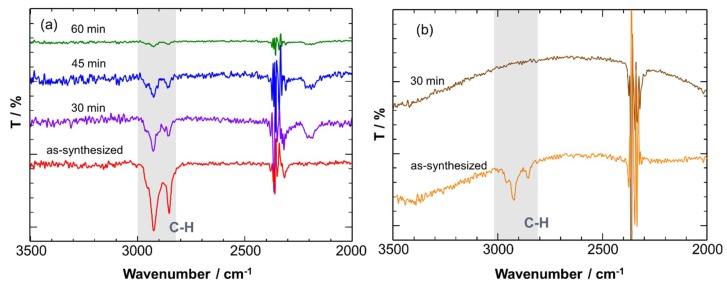
FT-IR spectra of (**a**) Cu_2_O and (**b**) CuO NCs heated at 200 and 250 °C, respectively, in air for different times.

On the basis of the above results, ligand stripping was carried out at 200 °C for 60 min in air. A lower treatment temperature is favorable to avoid the crystal growth. Figure 7a shows FT-IR spectra of the Cu_2_O NCs deposited on a Si substrate before and after heat treatment at 200 °C for different times. For an as-synthesized sample, diagnostic signals of oleylamine were seen at around 2900 cm^−1^ ascribable to symmetric and antisymmetric CH stretches. The intensity of the peaks gradually decreased with time and almost disappeared after 60 min, suggesting the removal of ligands from the NC surface. Notably, no change in the FT-IR spectra was seen at temperatures below 200 °C, suggesting that the surface ligands stably adsorbed on the Cu_2_O NCs at below 200 °C. On the other hand, a higher temperature treatment was employed for the CuO NCs because the CuO phase was stable at more than 250 °C, according to the XRD results. It was confirmed that surface ligands were completely removed from as-synthesized CuO NCs at 250 °C for 30 min, as shown in Figure 7b. From these results, the pre-treatment temperature was set to 200 and 250 °C for fabricating gas sensing layers composed of the Cu_2_O and CuO NCs, respectively.

### 3.3. H_2_S Sensing Properties of Copper Oxide NCs

It is well known that CuO-SnO_2_ gas sensors show very high sensitivity to H_2_S. Tamaki et al. proposed that the high sensitivity is due to the sulfurization of CuO to produce metallic CuS, which results in a significant change in the electrical resistance at the junction interface between CuO and SnO_2_ [7]. Since then, a large number of studies on CuO-SnO_2_ systems have been reported [17,19,45]. The H_2_S sensing ability for CuO has also been reported [46,47,48,49,50,51]. In this study, we examined the H_2_S sensing properties of the synthesized Cu_2_O and CuO NCs with an expectation that effective formation of electrical depletion layer in the NCs leads to high gas sensitivity.

**Figure 8:**
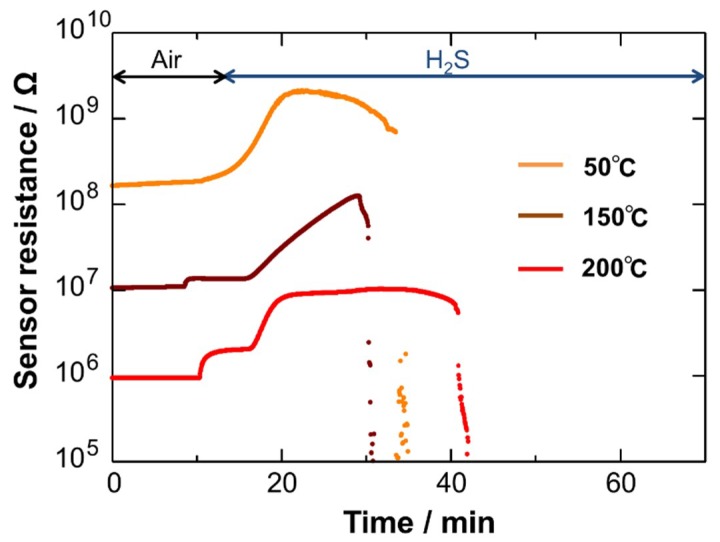
Response transients to 5 ppm H_2_S of the Cu_2_O NC sensor at different temperatures.

Figure 8 shows the electrical resistance of the Cu_2_O NCs in air and air containing 5 ppm H_2_S at different temperatures, such as 50, 100, and 150 °C. The resistance increased immediately after introduction of H_2_S. The increase in the resistance is a typical behavior for p-type sensor materials. The phenomenon observed for the p-type Cu_2_O NCs is explained as follows [52]. In air atmosphere, oxygen adsorption takes place on the Cu_2_O surface, expressed by the following reaction:

O_2_ + 2e^−^ → 2O^−^ (1)

The adsorbed oxygen extracts conduction electrons from Cu_2_O and forms an electron-depleted layer on the Cu_2_O NCs. The removal of carrier electrons leads to the accumulation of holes in Cu_2_O, decreasing its electrical resistance. Upon the introduction of H_2_S, the adsorbed oxygen reacts with H_2_S to release the trapped electrons according to the following surface reaction:

3O^−^ + H_2_S → H_2_O + SO_2_ + 3e^−^ (2)

The increase in electron concentration in Cu_2_O results in an increase in the probability of charge recombination between electrons and holes, leading to a decrease in hole concentration, and thus, increasing the electrical resistance.

However, the electrical resistance dropped after several minutes of the H_2_S introduction, as shown in Figure 8. This is obviously because sulfurization of Cu_2_O occurred to produce metallic Cu_2_S. This tendency was observed at all examined temperatures. The sulfurization of Cu_2_O and CuO with H_2_S proceeds as follows:

Cu_2_O + H_2_S → Cu_2_S + H_2_O (3)

CuO + H_2_S → CuS + H_2_O (4)

These reactions are thermodynamically feasible: the standard Gibbs free energies (Δ*G*^o^) of Equations (3) and (4) are −134.029 and −120.614 kJ/mol at 50 °C, respectively, and −135.542 and −121.377 kJ/mol at 150 °C, respectively. Utilizing their sulfurization by H_2_S, copper oxides have been used for H_2_S capture [53]. Thus, the results concluded that the native Cu_2_O NCs cannot be used for sensing H_2_S at low temperatures such as 50–150 °C.

**Figure 9:**
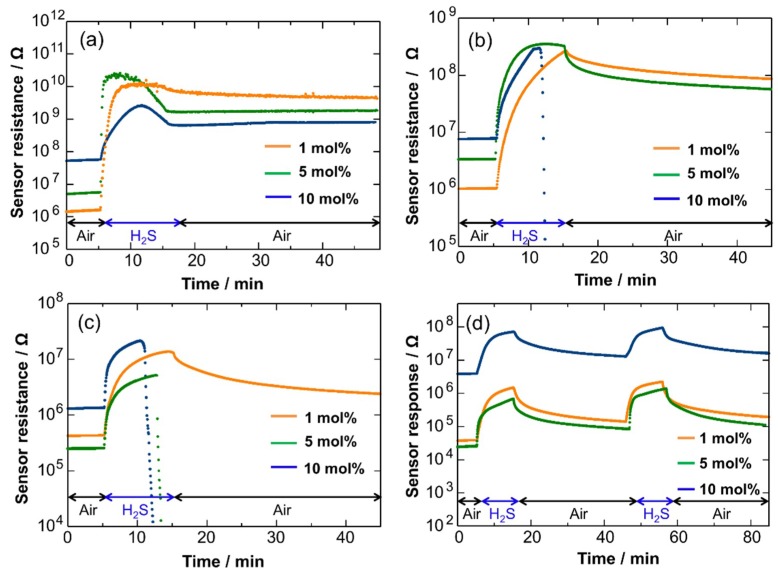
Response transients to 8 ppm H_2_S in air for Pd-loaded (1, 5, 10 mol%) Cu_2_O NC sensors at different temperatures. (**a**) 50, (**b**) 100, (**c**) 150, (**d**) 200 °C.

We next examined the gas sensing properties of Pd-loaded Cu_2_O NCs to 8 ppm H_2_S in air at 50–150 °C, as shown in Figure 9a–c. The electrical resistance was increased by Pd-loading, probably due to the formation of Schottky junctions between Pd and Cu_2_O. For a Pd (1 mol%)-loaded Cu_2_O sensor, no sudden drop in the electrical resistance was observed at all of examined temperatures (50–150 °C). However, for higher Pd-loading (5 and 10 mol%), the sensor resistance abruptly decreased, particularly at higher temperatures such as 100 and 150 °C. The results suggest that the loading of an appropriate amount of Pd on the Cu_2_O NCs suppresses the sulfurization of Cu_2_O and promotes the reaction of adsorbed oxygen with H_2_S. The promoting role of Pd for resistive-type gas sensors has been reported extensively in the literature [54]. The catalytic dissociation of H_2_S on Pd would facilitate the reaction of adsorbed oxygen with H_2_S. Another possible explanation is that Pd promoted the re-oxidation of sulfides, according to the following reactions:

Cu_2_S + 3/2O_2_ → Cu_2_O + SO_2_ (5)

CuS + 3/2O_2_ → CuO + SO_2_ (6)

These reactions are also thermodynamically favorable at the examined temperatures: the standard Gibbs free energies (Δ*G*^o^) of Equations (5) and (6) are −359.411 and −372.825 kJ/mol at 50 °C, respectively, and −350.166 and −364.33 kJ/mol at 150 °C, respectively.

**Figure 10:**
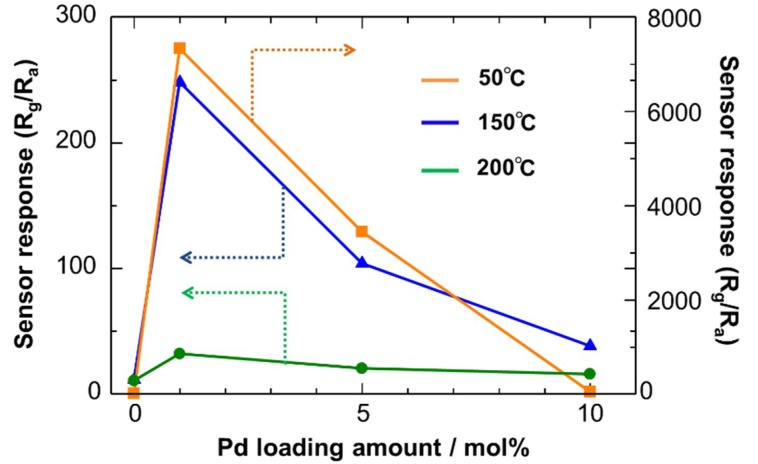
Dependence of sensor response to 8 ppm H_2_S in air on Pd loading amount for the Pd-loaded Cu_2_O NC sensors operated at different temperatures.

The dependence of sensor response (S = R_g_/R_a_) on Pd loading amount for the Cu_2_O NC sensors is depicted in Figure 10, which indicates the better promoting effect of Pd at lower temperatures. A significantly large sensor response was observed at 50 °C for the Cu_2_O NC sensor with 1 mol% of Pd loading. On the other hand, at higher temperatures, the promoting effect was not significant. One probable reason is that heterogeneous combustion of H_2_S occurred at the film surface at higher temperatures because of the highly-activated catalytic activity of the Pd NCs. This retards the diffusion of H_2_S inside the sensing film, limiting the surface reaction of H_2_S with adsorbed oxygen inside the sensing film and thus decreasing the sensor response. Such diffusion effects on sensor response have been experimentally and theoretically revealed for the detection of combustible gases using SnO_2_-based sensing films [55,56].

However, the complete recovery of the electrical resistance was not attained even for the Pd (1 mol%)-loaded Cu_2_O sensor at 50, 100, and 150 °C. The recovery took a long time, as shown in Figure 9a–c. The probable reason for this is slow re-adsorption of oxygen on Cu_2_O at lower temperature. Thus, the sensor properties were examined at 200 °C, although a part of Cu_2_O is converted to CuO at 200 °C, as shown in Figure 6. Figure 9d shows the response transient of the Pd-loaded Cu_2_O sensors at 200 °C. The sensors showed stable and reproducible responses to H_2_S, but the sensor response was decreased. The recovery speed was much improved compared with that at lower temperature, indicating the occurrence of reversible oxygen re-adsorption after switching the gas atmosphere from H_2_S in air to air. The good response and recovery behaviors of the Cu_2_O-CuO NCs are probably due to thermal activation of oxygen reaction and re-adsorption at higher temperature.

**Figure 11:**
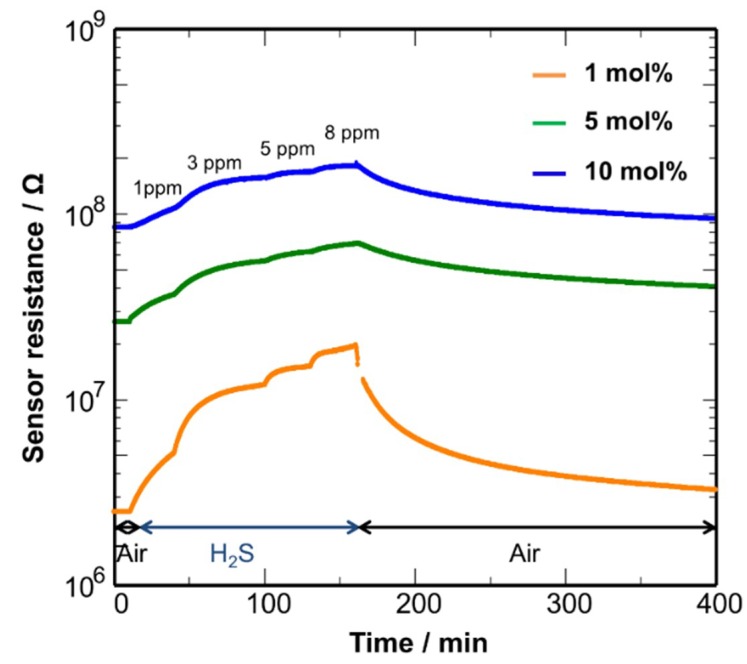
Response transients of the Pd (1, 5, 10 mol%)-loaded CuO NC sensor in response to 1–8 ppm H_2_S at 250 °C.

The sensing properties were also examined at 250 °C, as shown in Figure 11. At this temperature, Cu_2_O is entirely converted to CuO (Figure 6). The sensor response was reproducible: the resistance progressively changed with an increase in H_2_S concentration and almost recovered to the air base value after switching the gas atmosphere. The results indicate that the CuO NCs shows more favorable sensor responses at 250 °C. The Pd (1 mol%)-loaded CuO NCs showed the best performance with a quick response-recovery behavior, which should result from the activated catalytic activity of Pd NCs that were loaded with an appropriate amount. However, the 90% response time was 20 min. The modification of the microstructure of the sensing film is necessary to improve the diffusion rate of H_2_S. In contrast, the CuO NCs were not stable even with Pd loading at 50–150 °C, as shown in Figure S2. The electrical resistance sharply dropped upon H_2_S introduction, confirming the lower stability of the CuO NCs in a H_2_S atmosphere at 50–150 °C. Thus, it can be concluded that the sensors using the CuO and Cu_2_O NCs should be operated at more than 200 °C to avoid irreversible formation of CuS or Cu_2_S.

**Figure 12:**
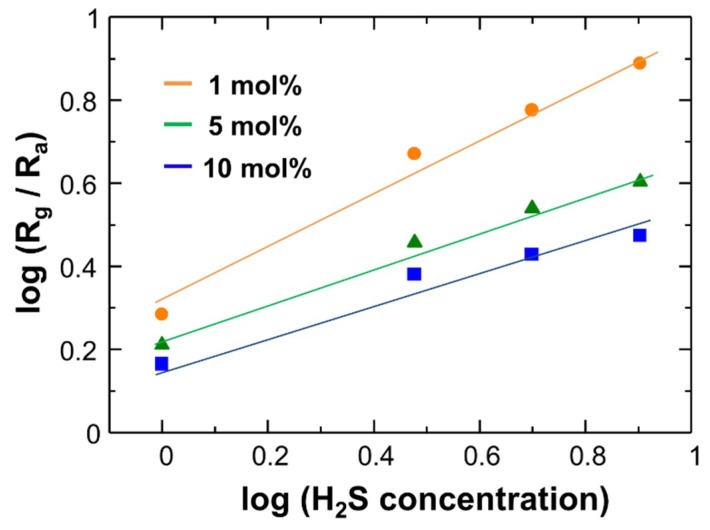
Dependence of sensor response on H_2_S concentration for the Pd (1, 5, 10 mol%)-loaded CuO NC sensors operated at 250 °C.

Figure 12 shows the dependence of the sensor response (S = R_g_/R_a_) on H_2_S concentration at 250 °C. The Pd (1 mol%)-loaded CuO NCs showed good sensor responses to H_2_S at diluted concentrations, reaching S = 7.9 for 8 ppm H_2_S. The obtained sensitivity is comparable to those for recently reported CuO sensors [57,58,59,60]. An effective formation of electron depleted regions in the CuO NCs by oxygen adsorption would lead to a large change in the electrical resistance after reaction with H_2_S. The sensor response is almost linear to the H_2_S concentration in a log-log scale, which is in accordance with a general trend in resistive-type gas sensors [61,62]. This suggests that the H_2_S sensing mechanism in Cu_2_O-CuO NC sensors is explained in terms of the reaction between adsorbed oxygen and H_2_S.

## 4. Conclusions

Cu_2_O and CuO nanocrystals (NCs) were synthesized by a heating method where copper (II) acetylacetonate was reacted with 1,8-octanediol in oleylamine at 160 °C. XRD analyses revealed the formation of Cu_2_O NCs with ca. 5 nm in crystallite size. It was found that the phase transformation of the Cu_2_O NCs to CuO NCs occurred by air oxidation at room temperature for a week. The phase conversion was also confirmed by UV-vis analyses, which clearly showed a change in the band gap of the Cu_2_O NCs after air oxidation. TEM and DLS analyses indicated the formation of monodispersed Cu_2_O and CuO NCs with ca. 12 and 24 nm in colloidal size, respectively. Gas sensing films were fabricated by spin coating using a colloidal suspension containing the Cu_2_O NCs. The thin film Cu_2_O sensor showed responses to H_2_S at 50–150 °C. However, the sensor signal suddenly dropped during measurements because of the formation of Cu_2_S upon reaction with H_2_S. In contrast, the loading of Pd NCs into the sensing film improved the stability of the Cu_2_O NCs. It is suggested that the Pd NCs would assist in the reaction of adsorbed oxygen with H_2_S and suppress the irreversible formation of Cu_2_S. To improve the recovery speed, the sensor was then operated at higher temperatures, which converted Cu_2_O to CuO. The Pd loaded-Cu_2_O/CuO sensor showed more reproducible responses to H_2_S at 200 and 250 °C, suggesting the occurrence of efficient oxygen re-adsorption. It is believed that deposition of Pd/Cu_2_O/CuO NCs on other semiconductors would further improve the H_2_S sensing performance, as demonstrated for ultrasensitive H_2_S detection in CuO/SnO_2_ systems.

## Supplementary Materials

The following are available online at http://www.mdpi.com/1424-8220/19/1/211/s1, Figure S1: XRD patterns of Cu_2_O NCs deposited on a Si substrate heated at 150 and 200 °C for different periods. Figure S2: Response transients to 8 ppm H_2_S in air for the Pd (1, 5, 10 mol%)-CuO NC sensors at different temperatures. (a) 50, (b) 100, (c) 150 °C.
