# Supplementary material for: Synthesis of Cu2O/CuO Nanocrystals and Their Application to H2S Sensing
Source: Sensors (Basel). 2019 Jan 8;19(1):211. doi: 10.3390/s19010211 (PMC6339020; doi:10.3390/s19010211)
Supplement: Supplementary file 1 [file sensors-19-00211-s001.pdf]

Supplementary Information

Synthesis of Cu_2O/CuO nanocrystals and their application to H_2S sensing

Kazuki Mikami [1], Yuta Kido [1], Yuji Akaishi [1], Armando Quitain [2], Tetsuya Kida [3]*

[1] Department of Applied Chemistry and Biochemistry, Graduate School of Science and Technology, Kumamoto University, Japan

[2] College of Cross-Cultural and Multidisciplinary Studies, Kumamoto University, Japan

[3] Faculty of Advanced Science and Technology, Kumamoto University, Japan

* Correspondence: tetsuya@kumamoto-u.ac.jp; Tel.: +81-96-342-3664 (T.K.)

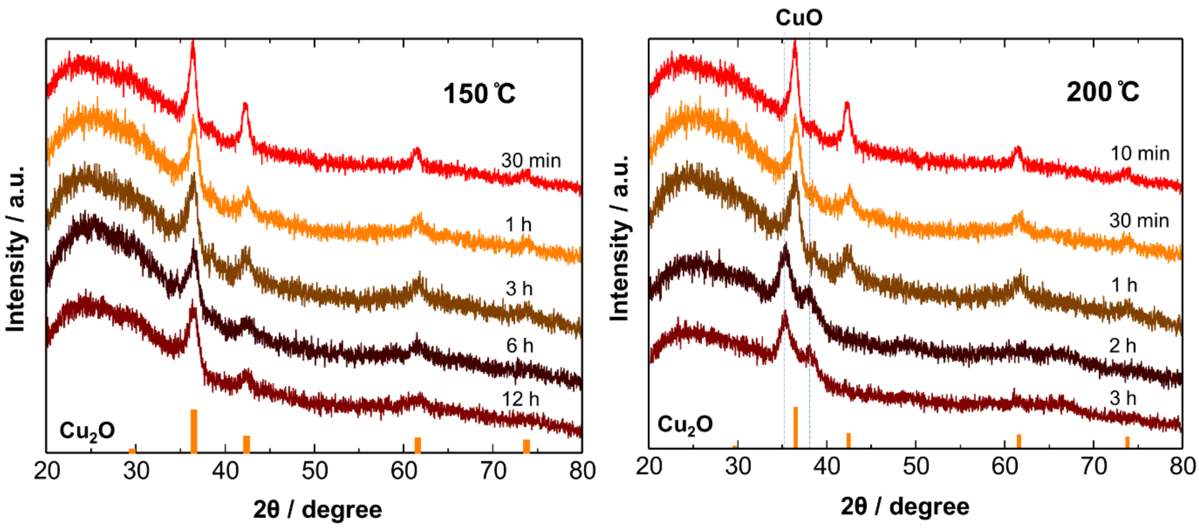

Figure S1. XRD patterns of Cu_2O NCs deposited on a Si substrate heated at 150 and 200°C for different periods.

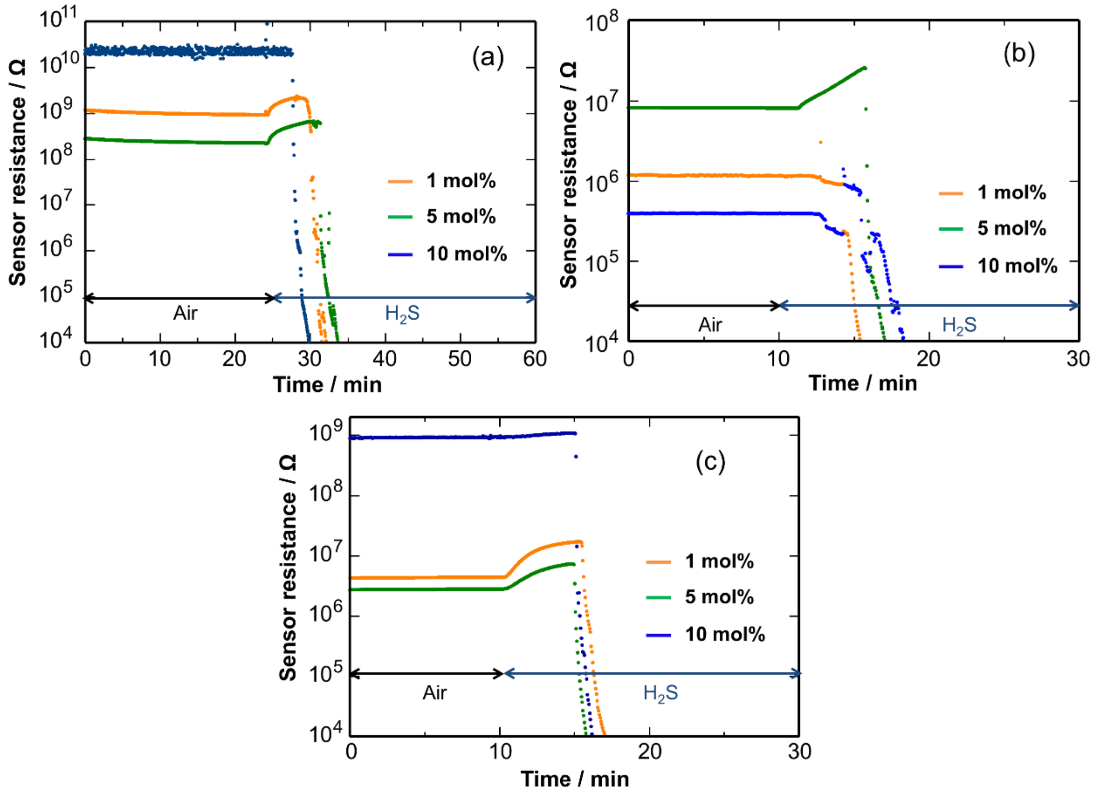

Figure S2. Response transients to 8 ppm H_2S in air for the Pd (1, 5, 10 mol%)-CuO NC sensors at different temperatures. (a) 50, (b) 100, (c) 150°C.
